# Nonlinear dynamics induced anomalous Hall effect in topological insulators

**DOI:** 10.1038/srep19803

**Published:** 2016-01-28

**Authors:** Guanglei Wang, Hongya Xu, Ying-Cheng Lai

**Affiliations:** 1 School of Electrical, Computer, and Energy Engineering, Arizona State University, Tempe, AZ 85287, USA; 2 Department of Physics, Arizona State University, Tempe, AZ 85287, USA

## Abstract

We uncover an alternative mechanism for anomalous Hall effect. In particular, we investigate the magnetisation dynamics of an insulating ferromagnet (FM) deposited on the surface of a three-dimensional topological insulator (TI), subject to an external voltage. The spin-polarised current on the TI surface induces a *spin-transfer torque* on the magnetisation of the top FM while its dynamics can change the transmission probability of the surface electrons through the exchange coupling and hence the current. We find a host of nonlinear dynamical behaviors including multistability, chaos, and phase synchronisation. Strikingly, a dynamics mediated Hall-like current can arise, which exhibits a nontrivial dependence on the channel conductance. We develop a physical understanding of the mechanism that leads to the anomalous Hall effect. The nonlinear dynamical origin of the effect stipulates that a rich variety of final states exist, implying that the associated Hall current can be controlled to yield desirable behaviors. The phenomenon can find applications in Dirac-material based spintronics.

Hall effect is one of the most striking and widely investigated phenomena in contemporary physics. The classical Hall effect is simply due to the Lorentz force, while the quantum Hall effect can be attributed to the emergence of surface states due to the formation of Landau levels in a magnetic field, and the fractional quantum Hall effect results from many-body interactions^1^. In certain materials, without any magnetic field, the spin-orbit interaction can lead to spin Hall effect, the physical base for topological insulators (TIs)^2^,^3^,^4^, an area of tremendous recent interest in condensed matter physics. In this paper, we report our finding of an alternative mechanism, a mechanism that is essentially dynamics based, which can result in an anomalous Hall effect. The underlying physics is spin-transfer torque.

Spin-transfer torque originates from the exchange coupling between the magnetisation in a ferromagnet and a polarised spin current. When such a current flows close to the ferromagnet, a finite torque will be exerted on the magnetisation of the ferromagnet, provided that the magnetisation vector is not aligned with the direction of spin polarisation. Semiclassically, the dynamical evolution of the magnetisation can be described by the Landau-Lifshitz-Gilbert (LLG) equation^5^. In spintronics applications, spin-transfer torque can be quite useful as it provides a way to manipulate or even switch, electronically, the magnetisation in the ferromagnet, which can lead to reduced dimensions and efficient energy consumption as compared with conventional magnetic schemes^6^. Previous studies on spin-transfer torque focused on heavy metals with strong spin-orbit coupling, which can generate significant spin-polarised current through the spin Hall effect^7^,^8^,^9^ or in materials with strong Rashba spin-orbit coupling effect at the interfaces^10^,^11^,^12^,^13^. Another promising material is TIs that possess a bulk band gap but with metallic massless Dirac surface states^2^,^3^,^4^. The strong Rashba-type SOC guarantees that the momentum of the surface electron is interlocked with its spin. When a TI is coated with a thin film of insulating ferromagnet, a host of novel magnetoelectric effects can arise. In this configuration, phenomena that have been predicted theoretically include the inverse spin-Galvanic effect^14^, current-induced magnetisation reversal^15^, anomalous magnetoresistance of a two-dimensional ferromagnet/ferromagnet junction^16^, and auto-oscillations of magnetisation^17^.

Here, we focus on the dynamics of magnetisation in the insulating ferromagnet as well as the spin-polarised current on the surface of TI. When the system is subject to a periodic electric driving signal with a *dc* offset applied to the the TI surface, rich nonlinear dynamical phenomena in magnetisation can arise in the upper insulating ferromagnet via the induced spin-transfer torque, which include multistability, chaos, and phase synchronisation. In certain range of the ratio of the *dc* and *ac* amplitudes, e.g., [−1, 1], there are critical points at which the magnetisation dynamics in the ferromagnet can change abruptly between two stable states (attractors), leading to adiabatic dynamical transport of the surface electrons in the 3D TI via the exchange coupling (i.e., the proximity effect). Our main finding is the emergence on the surface of the TI of an unconventional Hall-like current resulting from the spin-transfer torque induced magnetisation. This type of Hall effect is characterised by a multivalued functional relation between the transverse and longitudinal conductance. The nonlinear dynamical mechanism uncovered here represents an alternative route to Hall effect. The dynamics-induced anomalous Hall effect can have potential applications in spintronics.

## Model

**Figure 1:**
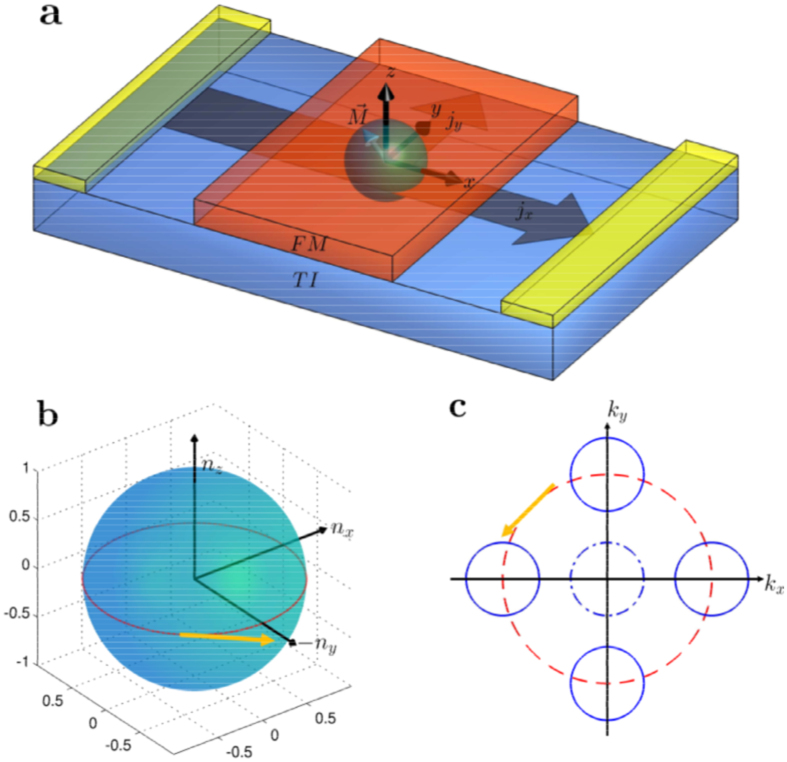
Schematic illustration of the TI-FM coupled system and the basic interactions. (**a**) A thin layer of insulating ferromagnet sits on a 3D TI. A voltage is applied along the *x* direction. The effective magnetization of the ferromagnet, ***m***, oscillates in time and is coupled to the spin-polarized currents 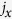 and 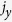 in the TI through the mechanism of exchange coupling. (**b**) A typical time evolution pattern of the normalized magnetization ***n***. (**c**) Change in the position of the Fermi surface in the wave vector space associated with the surface electrons of the TI, which corresponds to the adiabatic evolution of ***n*** in (**b**). The central dashed circle corresponds to the case where the ferromagnet is absent.

We consider a coupled system consisting of a ferromagnet and a 3D TI, as shown in Fig. 1, where the former sits on the top surface of the latter. When a voltage is applied to the surface, a spin-polarised current is induced along the direction of the voltage drop, i.e., the +*x* direction, which is the easy axis of the upper ferromagnet (an energetically favorable direction of spontaneous magnetisation). As the spin-polarised current flows through the region of the ferromagnet, the net spin will exert a torque, i.e., spin-transfer torque, on the magnetisation of the ferromagnet, the dynamics of which is governed by the LLG equation. The exchange coupling between the two materials will change the transport behaviors of the TI, leading to a redistribution of the spin-polarised current. The typical time scale of the dynamical evolution of magnetisation is on the order of 1 ns, which is quite slow as compared to the surface electron response time. It is thus reasonable to apply the adiabatic approximation to modeling the dynamical behavior of the surface electrons. In particular, the transmission coefficient of the surface electron of the TI can be obtained by solving the time-independent Schrödinger equation with a constant exchange coupling term at any specific time^15^,^17^. The low energy effective surface state Hamiltonian of the TI is given by


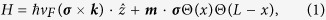


where 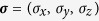 are the Pauli matrices describing the spin of the surface electron, 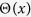 and 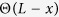 are step functions to ensure that only the ferromagnet region (0 < *x* < *L*) has the exchange coupling, 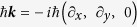 is the momentum operator for the surface electron, 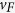 is the Fermi velocity, and ***m*** is the magnetisation in the upper ferromagnetic layer. We assume the surface of the TI is within the 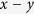 plane so that the *z* component of the surface electron momentum is zero, as shown in Fig. 1a. The first term of the effective Hamiltonian describes the conventional spin-orbit coupled TI surface state while the second term introduces the electron exchange interaction with the proximate ferromagnet. Let 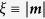 be the magnitude of the vector ***m*** so that 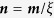 is the unit vector of magnetisation. A typical evolution of ***n*** is shown in Fig. 1b.

From the Hamiltonian, we can calculate the transmission coefficient through the ferromagnet region. The procedure is to write down the wavefunctions before entering, inside and after exiting the ferromagnet region, and then to apply the boundary conditions at the interfaces of the three regions. The result is^15^


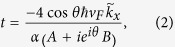


where


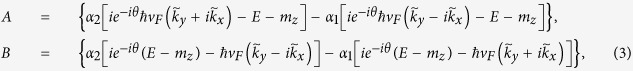



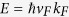
, 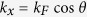, 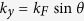, 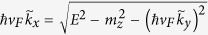, 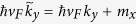, 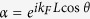, 
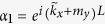
, and 
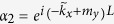
. Here 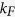 is the Fermi wave vector and *θ* is the incident angle of the electron to the ferromagnet region in the TI. From the transmission coefficient *t*, we can get the current densities along the *x* and *y* directions as


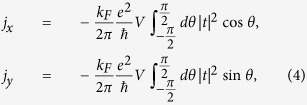


where 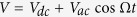 is the driving voltage in the *x* direction. On the surface of a TI, the spin density of the electrons can be written as


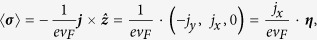


where 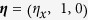. The quantity 
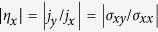
 is the ratio of the Hall conductance to the channel conductance^17^,^18^. Indeed, when the ferromagnet is absent, we have


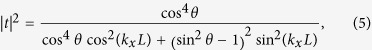


which is an even function of *θ* so that 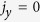. However, under the influence of the magnetisation, 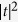 is no longer an even function of *θ*, which leads to a finite 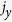. Physically, this is because the *y* components of the current contributed by the electrons with incident angles *θ* and 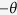 do not have the same magnitude so that a net 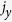 component appears. The situation can be seen schematically in Fig. 1c, where the central dashed circle is electron’s Fermi surface in the momentum space without the effect of the magnetisation. However, when a current flows through the ferromagnet, the magnetisation evolves [Fig. 1b] and the position of the Fermi surface will shift, as shown in Fig. 1c. Since there is no external voltage applied along the *y* direction, 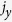 originates from its interplay with 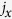 through the exchange coupling, giving rise to an anomalous Hall effect.

When a spin-polarised current flows in the TI under the ferromagnet, the dynamics of the magnetisation is governed by the following LLG equation:


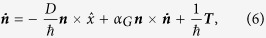


where the first term describes the procession along the easy axis 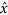 and *D* represents the anisotropy energy of the ferromagnet. The second term characterises the damping of the magnetisation with 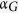 being the Gilbert damping constant. The last term represents the spin-transfer torque 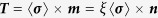, which comes from the effective exchange coupling averaged over the entire ferromagnet region.

In our simulations, we set the initial direction of the magnetisation to be along the easy axis, i.e., 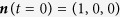. We then calculate the electron transmission coefficient and the average spin density. The magnetisation at the next time step can be obtained through the updated spin-transfer torque.

## Results

### Bifurcation diagram and anomalous Hall currents

**Figure 2:**
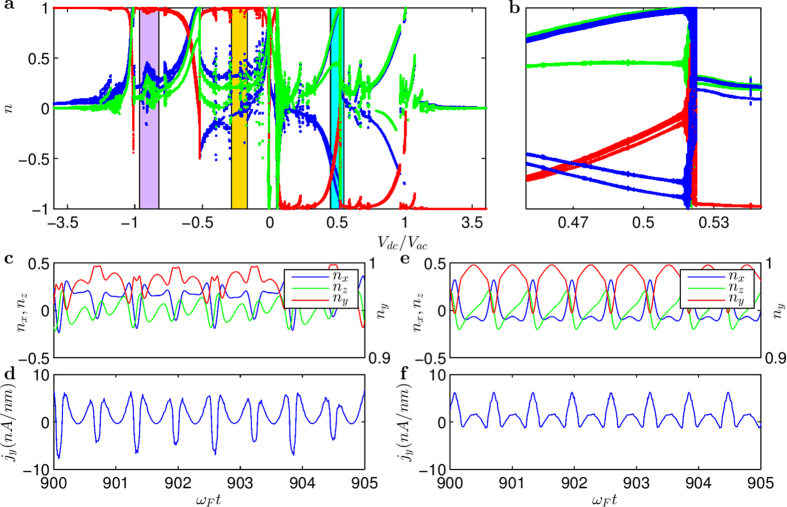
Typical dynamical behaviors of the TI-FM coupled system. (**a**) Bifurcation diagram of the magnetization with respect to ratio of applied dc and ac voltages. Blue, red and green dots correspond to *n_x_*, *n_y_*, and *n_z_*, respectively. One chaotic regime is marked by a purple stripe and one phase-synchronization regime is marked by a yellow stripe. (**b**) Magnification of the transitional region in the bifurcation diagram in (**a**) (marked with cyan strip). (**c**) A representative time series of the magnetization from the purple chaotic regime. (**d**) Anomalous Hall current for a parameter value in (**c**). (**e**) A representative time series of the magnetization from the yellow phase synchronization regime, and (**f**) the corresponding anomalous Hall current. For better visualization, the *x* axis in (**a**) is rescaled.

The coupling term in Eq. (6) is nonlinear, rendering possible complex dynamical behaviors including chaos. These behaviors can be uncovered through a systematic computation of the bifurcation diagram. Specifically, we set 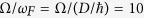, 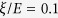, 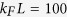, 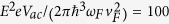, and use the ratio 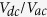 as a convenient bifurcation parameter (in the range [−4, 4]). The resulting bifurcation diagram is shown in Fig. 2a. We observe a rich variety of dynamical behaviors, e.g., a chaotic regime (marked with purple stripe) and a phase synchronisation^19^ region (marked with yellow stripe). Representative time series in the chaotic regime are shown in Fig. 2c,d, and those from the phase synchronisation region are shown in Fig. 2e,f. Considering that the driving frequency Ω is ten times larger than the intrinsic procession frequency of magnetisation, it is remarkable that phase synchronisation can occur. From the behavior of *n_y_* in Fig. 2e, we see that it undergoes periodic oscillations of period about 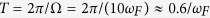.

The time series shown in Fig. 2d,f are in fact the time evolutions of the anomalous Hall current density 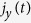. For Fig. 2d, the behavior is chaotic, which can be seen by noting, e.g., that the occurrences of the local minima and their values are irregular. For Fig. 2f, the current density exhibits a period-2 behavior. In both cases, the rms (root mean square) value of the Hall current density is finite. The Hall effect is anomalous because it is a result of dynamical interactions between the magnetisation in the FM and the surface electron motion in the TI. As we argue later, it has a dynamical origin, i.e., it is induced by the nonlinear, dynamical interactions mediated by the spin-transfer torque between the FM and TI.

### Multistability

From the bifurcation diagram in Fig. 2, we see that there are a number of transitional regions, in which the dynamics of the system change characteristically. Figure 2b is a magnification of one transitional region, marked with the cyan strip in Fig. 2a for 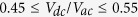. We see that there exists a rather abrupt change in the bifurcation behavior about 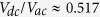. Generally, a transitional region along the parameter axis is one in which there is multistability. Preceding the transition, there is one final state (attractor). At the onset of the transition, a new attractor is born, leading to coexistence of two attractors, each with its own basin. As the parameter is varied (e.g., increased) in the transitional region, the basin of the new attractor grows while that of the “old” attractor, the continuation of the attractor before the transition, shrinks. The disappearance of the basin of the “old” attractor marks the end of the transitional region. Thus, before and after the transition, the final states of the system are typically characteristically distinct.

**Figure 3:**
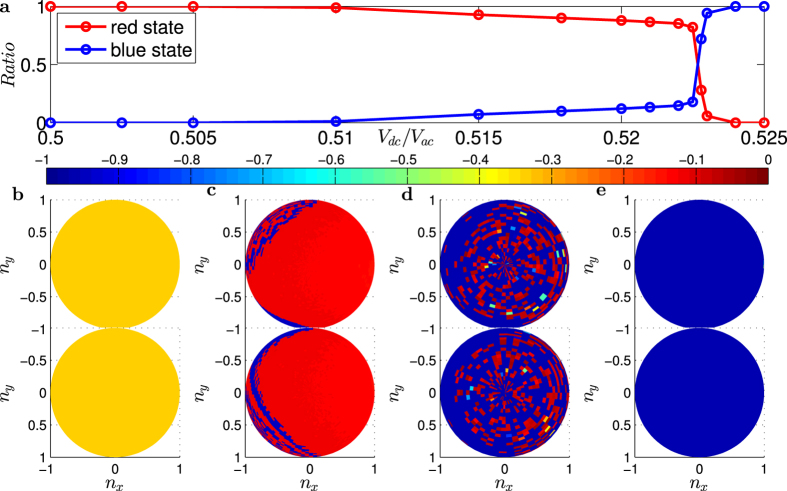
Final states (attractor) before and after transition. (**a**) Change in the ratio of the red states and states with system parameter 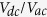 in the transitional region. (**b–e**) Basins of attraction of the red and blue states for *V*_*dc*_/*V*_*ac*_ = 0.5, 0.5179, 0.5228 and 0.525, respectively. Each initial condition is assigned the color of the final state that it leads to, which can be conveniently distinguished by the mean value of 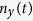.

To obtain a comprehensive picture of the dynamical evolution of the TI-FM coupled system in the transitional region, we use systematically varying initial conditions. The results are shown in Fig. 3. Since the magnetisation vector ***n*** has unit length, the phase space is effectively the surface of a unit sphere in which all possible initial conditions and final states lie, where the initial conditions can be represented as 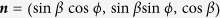. Choosing a uniform, 100 × 100 grid for 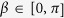 and 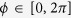, we obtain 10^4^ different initial conditions and calculate the final attractor for each initial condition. To distinguish the final states from different initial conditions, we calculate the mean value of 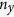 after a relative long evolution time. Figure 3c shows a typical basin of attraction for 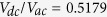, where the upper half part is an aerial view of the unit sphere while the bottom half is a ground view. Referring to the color bar we see that there are two distinct final states: a red state with *n*_*y*_ ≈ 0 and a blue state with *n*_*y*_ ≈ −1. Figure 3a shows the fraction of the red/blue states versus the bifurcation parameter, where the relative percentages of both states change monotonically. Figure 3b–e show four basins of attraction for 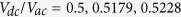 and 0.525, respectively. These results suggest the following general scenario for the occurrence of multistability. As a parameter changes, a transitional region emerges, where the beginning of the region is marked by the appearance of a new attractor. As the parameter varies into the transitional region, the basin area on the unit sphere of the old attractor decreases but that of the new attractor increases. Termination of the transitional region is associated with the complete disappearance of the old attractor.

**Figure 4:**
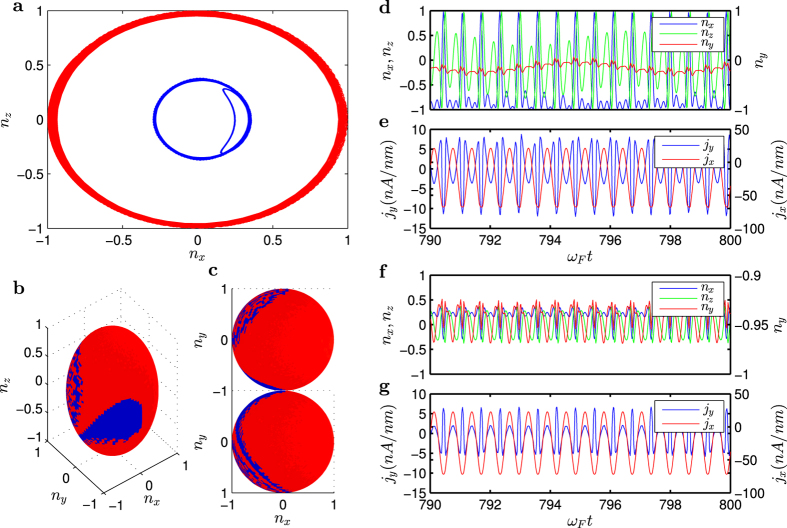
Multistability. (**a**) For *V*_*dc*_/*V*_*ac*_ = 0.5179 in the transitional region, two coexisting final states (red and blue attractors) in the *n_x_* – *n_z_* phase space. (**b**) The corresponding initial conditions that lead to the attractors in (**a**). (**c**) A two-dimensional view of (**b**), where the upper panel is an aerial view and the lower panel is a ground view. (**d**) Typical time series of the magnetization for the red state. (**e**) Typical time series of the anomalous Hall and channel current densities for the red state. (**f**) Typical time series of the magnetization for the blue state. (**g**) Typical time series of the anomalous Hall and channel current densities for the blue state. Note that the ranges of variation in 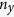 are different in (**d**,**f**), providing a convenient criterion to computationally distinguish the red state from the blue state.

In nonlinear dynamical systems, multistability is a common phenomenon^20^,^21^,^22^,^23^,^24^,^25^. We now present direct evidence of multistability in the transitional region for the TI-FM coupled system. Figure 4a shows, for 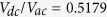, two distinct trajectories projected into the *n_x_* − *n_z_* plane, which correspond to two different final states (colored according to the mean value of *n_y_* after disregarding reasonably long transients. Figure 4b shows the corresponding basins of attraction, and Fig. 4c shows two-dimensional views of Fig. 4b (aerial and ground views). Specifically, one final attractor has near zero mean *n_y_* values (colored with red). For this attractor, the magnetisation oscillates in the vicinity of the 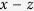 plane, and both *n_x_* and *n_z_* exhibit a dramatic switching behavior between the extreme values −1 and 1, as shown in Fig. 4d. (The corresponding time series of distinct current densities are shown in Fig. 4e–to be discussed below.) For the blue attractor, the mean *n_y_* value is close to unity so that the magnetisation vector is fixed about *n*_*y*_ = −1 with small oscillations in *n_x_* and *n_z_*. That is, the magnetisation vector is oriented close to the −*y* direction, as shown in Fig. 4f. Depending on the initial condition, the system settles into either the red or the blue attractor, signifying multistability.

### Anomalous Hall effect

A remarkable phenomenon is that, about the transitional region, there is current in the direction transverse to the channel, i.e., 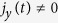. This is essentially the Hall current density induced by the coupling dynamics between FM and TI. The anomalous Hall current is persistent in that it exists prior to, during, and after the transition. As the transitional region is swept through, there is a phase change associated with the Hall current. In particular, for 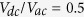 (before transition), the oscillations of the Hall current density are in phase with the external electrical driving, as shown in Fig. 4e. For 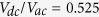 (after transition), the Hall current is 180° out of phase with the driving, as shown in Fig. 4g. During the transition, the two coexisting states (attractors) retain the respective phase relations with the driving. For example, as shown in Fig. 4e, for 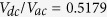, the red attractor has the same phase as the external driving, which is the continuation of the attractor before the transition. However, as shown in Fig. 4g, the blue attractor, which evolves to become the attractor after the transition, has the opposite phase to that of the driving.

To gain insights into the physical and dynamical origin of the anomalous Hall effect in the TI-FM coupling system, we examine the dependence of the Hall conductance, 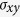, on the channel conductance 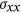, which are defined as


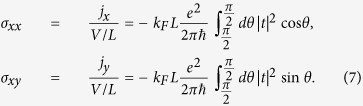


**Figure 5:**
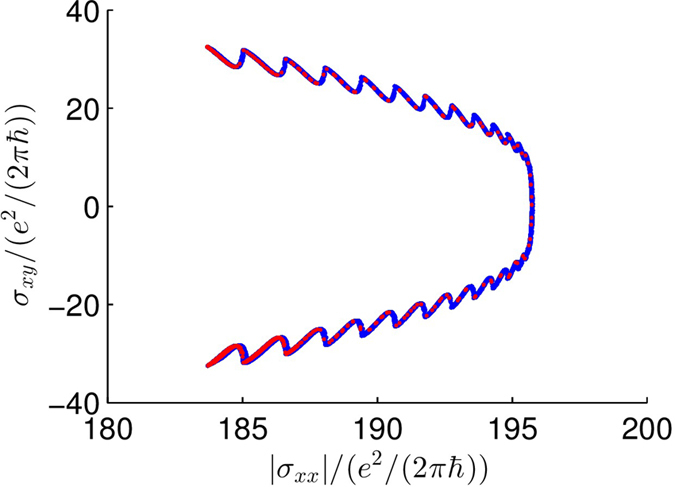
Dependence of the anomalous Hall conductance on channel conductance. For 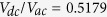 (in the transitional region) and the red attractor, the normalized Hall conductance versus the normalized channel conductance, where the blue dots are results from 100 periods of oscillation and the red dots are results from one oscillating period. The functional relation between 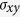 and 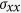 is multi-valued (see Fig. 6 for a dynamical explanation.

Some representative results are shown in Fig. 5 for 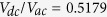 (in the transitional region) for the red attractor, where the initial condition is 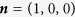. Similar results have been found for the blue attractor. In previous studies where an anomalous Hall effect arises, the Hall conductance is typically positively correlated with the channel conductance^18^, e.g., 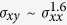. However, in our TI-FM coupled system, the correlation is negative. From Eq. (7), we see that the total current is divided into the *x* and *y* components through the functions cos*θ* and sin*θ*, where the quantity 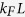 has a physical meaning of the number of available carriers within the ferromagnetic region, the integrals characterise the probabilities of transporting to the *x* or *y* directions for a single carrier, and 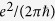 is the fundamental conductance unit. Because of the small probability of backscattering, if more electrons transport along the *x* direction, there will be fewer electrons transporting in the *y* direction, and vice versa. This behavior can be seen from Fig. 5, where we have 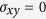 when 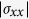 is maximal. Without FM, i.e., for a bare TI, the quantum transmission 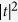 is an even function of *θ*, leading to zero Hall current. When FM is present, the dynamical coupling between its magnetisation and channel current in the TI induces a transverse, Hall current, making time-dependent the relative distribution of the electrons in both directions.

**Figure 6:**
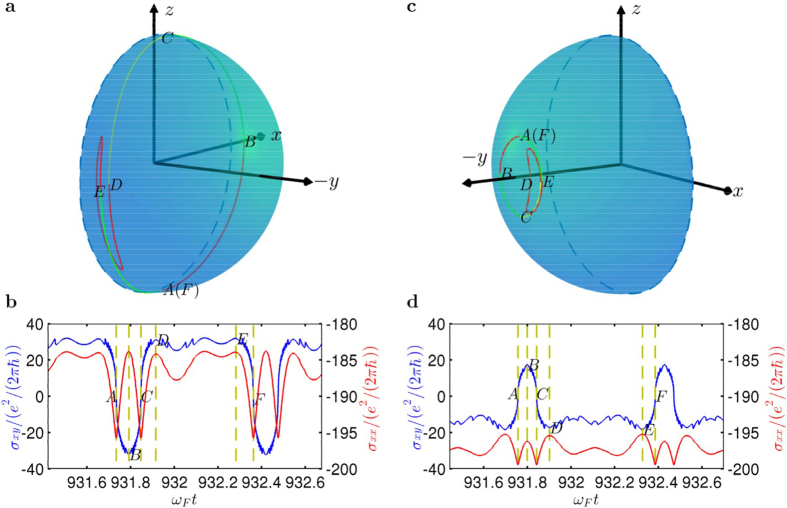
Dynamics of magnetization in the multistability regime and its relation with the surface currents. (**a**) For 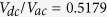 in the transitional region, evolution of the magnetization of the red attractor (continuation of the attractor preceding the transition) within one driving period, where the initial condition is 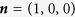. (**b**) The corresponding time series of the Hall and channel conductance within the same time period. (**c**) For 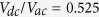 (after transition), evolution of the magnetization vector for the blue attractor. (**d**) The corresponding Hall and channel conductance within the same time period. The letters 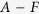 mark the local maxima and minima of the channel conductance.

Figure 5 indicates that, the Hall versus the channel conductance is a multivalued function: for one channel conductance value there are two values of the Hall conductance. The function is symmetrical with respect to the line 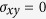. To understand these features, we examine the relation between magnetisation in the FM and the surface currents in the TI. Figure 6 demonstrates the detailed dynamical behaviors of system for two parameter values: 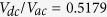 (during the transition) and 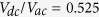 (after the transition), where Fig. 6a shows the evolution of the magnetisation in the FM for one driving period and Fig. 6b shows the currents during the same time period. Each section in Fig. 6a, marked with a different color, corresponds to a section from a local maximum (minimum) to a local minimum (maximum) of the channel conductance in Fig. 6b, as marked by the symbols 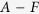. The attractor in Fig. 6a has its magnetisation oscillating close to the 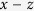 plane (the red attractor in Fig. 4). A careful examination of the detail of the dynamical evolution indicates that, when 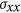 reaches its maximum (marked as 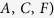, the Hall conductance 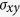 is close to zero, and the *x* component of the magnetisation is nearly zero as well. This is a consequence of the interaction in the TI-FM coupled system. In particular, Eq. (1) stipulates that the *y* component of the electron momentum is coupled to the *x* component of the spin. Due to the exchange interaction between the magnetisation and the current spin, the electron spin tends to align with the magnetisation to minimise the total energy. For 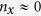, the electrons tend to polarise their spin along other directions, making 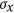 close to zero so that the *y* component of the current becomes vanishingly small. For 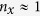 (marked with 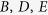 in Fig. 6a), the spin of the electrons tends to polarise along the *x* direction, resulting in a very large *y* component of the current, as shown in Fig. 6b. There is thus a strong correlation between the direction of the magnetisation in the FM and the current direction in the TI.

To explain the symmetry of 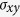 in Fig. 5, we examine the dynamics of the system about the points *A* and *C* in Fig. 6b, where 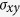 and 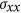 are approximately an odd and an even functions of time, respectively. As a result, for a particular value of 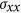, there exists two values of 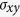 with opposite signs and equal magnitude, which correspond to the two different colored sections around *A* and *C*, as shown in Fig. 6a. We also note that, Fig. 6b exhibits a plateau region of the Hall conductance, i.e., during the time interval between *D* to *E*. This is due to a reverse behavior of the magnetisation about the −*x* direction, as marked by the red section in Fig. 6a.

The above analysis can be readily extended to the coexisting state (the blue attractor in Fig. 4), as shown in Fig. 6c,d. The reason for a relatively small Hall current in this case is that the magnetisation is confined along the −*y* direction so that the value of 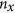 is relatively small. The plateau behavior of the Hall conductance is opposite to that in Fig. 6b because the reversed motion lies in the opposite arc of the magnetisation sphere.

## Discussions

The exchange coupling between magnetisation (e.g., in a FM) and the polarised spin current (e.g., in a TI) can lead to surprising physical phenomena. The dynamics of such coupled quantum systems can be treated semiclassically through the LLG equation^5^, which are generically nonlinear. As a result, rich nonlinear dynamical behaviors can arise in the system, including multistability, chaos, and phase synchronisation.

The main accomplishments of this paper are twofold: from the perspectives of nonlinear dynamics and physics. Dynamically, we focus on the phenomenon of multistability through a detailed investigation of bifurcation and system’s behaviors in the transitional regions. In particular, we demonstrate that characteristic changes in the system’s final state (or attractor) is associated with the emergence of multistability in the transitional region. Prior to the region, the system exhibits one attractor. As the system enters into the transitional region, a new attractor is born, with its own basin of attraction. Further into the transitional region, the basin of the new attractor expands, while that of the original attractor is suppressed at the same time. At the end of the region, the basin of the original attractor vanishes, and that of the “new” attractor dominates the entire phase space, completing the transition process and leading to a characteristically different attractor.

Physically, we uncover a novel type of Hall effect: due purely to nonlinear dynamical interactions a current transverse to the channel current on the surface of the TI can arise. The Hall current exhibits a nontrivial dependence on the channel current. The phase of the Hall current relative to that of the electrical voltage depends on the final state of the system and can exhibit a change of *π* as the system parameter changes through the transitional region. For example, before the transition the system falls into an attractor, associated with which the Hall current is in phase with the electrical driving. The new attractor created in the transitional region has the property that the phase of the associated Hall current is opposite to that of the driving. As a result, after the transitional region is passed, the phase difference between the Hall current and the electrical driving becomes *π*. Another feature is that, the anomalous Hall current has a negative correlation with the channel current, which can again be understood through the nonlinear dynamics of the magnetisation in FM.

To our knowledge, the dynamics induced anomalous Hall effect through the spin-transfer torque uncovered in this paper is a novel phenomenon. The underlying coupled system between FM and TI can be realised in experiments. A set of experimentally feasible parameter values can be, e.g, 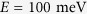 and 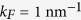, leading to 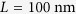 and 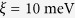, where we assume 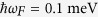 and 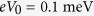. An appealing feature of this Hall effect is that the dynamical properties of the Hall current can be controlled readily through the channel voltage, e.g., by modulating the ratio between its ac and dc components. The fact that the whole system can exhibit a rich variety of nonlinear dynamical behaviors means equally many possibilities to realise the Hall current. This has potential applications in spintronics.

*Notes:* During an inquiry process with a physics journal, the editor brought to our attention a recent paper^26^ that reported anomalous Hall effect in a similar setting. In particular, the paper studied the nonlinear dynamics of the magnetisation in the FM insulator deposited on a TI surface. Considering both ballistic, non-coherent and diffusive transport regimes under a dc driving, the authors found anomalous Hall current in both regimes and its effects on the final magnetisation states. In our work, we employed the conventional coherent treatment of spin-polarised current transport (i.e., in the ballistic regime) and focused on the nonlinear dynamics under *both* ac and dc driving. We obtained, quantitatively, the dependence of the anomalous Hall conductance on the magnetisation and uncovered, strikingly, an unconventional dependence on the channel conductance. We also found the phenomenon of multistability and fully explained its emergence and evolution. In addition, we demonstrated that the STT intermediated FM-TI coupled system can exhibit a rich variety of nonlinear dynamical phenomena.

## Additional Information

**How to cite this article**: Wang, G. *et al*. Nonlinear dynamics induced anomalous Hall effect in topological insulators. *Sci. Rep.* **6**, 19803; doi: 10.1038/srep19803 (2016).
